# Complete Response to R-EPOCH in Primary Cardiac Lymphoma

**DOI:** 10.1155/2019/7690430

**Published:** 2019-04-10

**Authors:** Kartik Anand, Sai Ravi Pingali, Barry Trachtenberg, Swaminathan Padmanabhan Iyer

**Affiliations:** ^1^Houston Methodist Cancer Center, Weill Cornell Medicine, Houston, TX, USA; ^2^Division of Cardiology, DeBakey Heart & Vascular Institute, Houston Methodist Hospital, Houston, TX, USA; ^3^Department of Lymphoma/Myeloma, UT MD Anderson Cancer Center, Houston, TX, USA

## Abstract

Primary cardiac lymphoma (PCL) is a rare extranodal lymphoma involving only the heart and/or the pericardium. Most common presenting signs and symptoms are nonspecific including dyspnea, pericardial effusion, and arrhythmia. Prognosis of PCL patients remain poor compared to non‐cardiac lymphoma patients. Since most of the information about PCL comes from case reports or case series, there is no treatment consensus. Anthracycline containing chemotherapy remains main treatment modality which is potentially cardiotoxic. We present a case of PCL that achieved complete remission using R-EPOCH (rituximab, etoposide, prednisone, vincristine, cyclophosphamide, and doxorubicin). We also used dexrazoxane in an effort to reduce cardiotoxicity of chemotherapy.

## 1. Introduction

Primary cardiac lymphoma (PCL) is a rare malignancy, accounting only for 1.3% of cardiac tumors and 0.5% of extranodal lymphoma [1]. PCL was first described in a German publication in 1939 [2]. PCL is defined by the WHO as an extranodal lymphoma, involving only the heart and/or the pericardium [3]. Most common presenting signs and symptoms of PCL are dyspnea, arrhythmia, congestive heart failure, and pericardial effusion, as these signs and symptoms are nonspecific, making prompt diagnosis of PCL challenging. Before the advent of cardiac imaging (e.g. echocardiography), most cases were diagnosed at autopsy. Likely due to the increased frequency of cardiac imaging, more cases are being diagnosed each year [4]. The most common histologic subtype for PCL is diffuse large B-cell lymphoma [5]. Currently, there is no treatment consensus for PCL due to its rare presentation, and most of the literature comes from case reports. Anthracycline containing chemotherapy remains the most frequently used treatment modality [4]. SEER database analysis shows that median overall survival for cardiac lymphoma patients remains poor compared to non‐cardiac lymphoma patients [5]. Herein, we report a case of PCL with treatment using dose adjusted R-EPOCH (rituximab, etoposide, prednisone, vincristine, cyclophosphamide, and doxorubicin) which leads to complete remission (CR). We also explore other interesting clinical considerations in this case, including the use of dexrazoxane in the treatment of PCL.

## 2. Case Presentation

**Figure 1:**
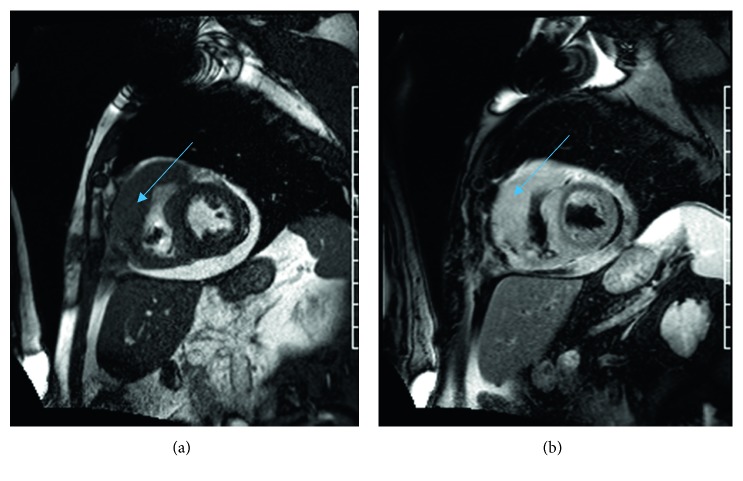
Cardiac MRI showing mass in lateral wall of the left ventricle on T1 and T2 imaging.

**Figure 2:**
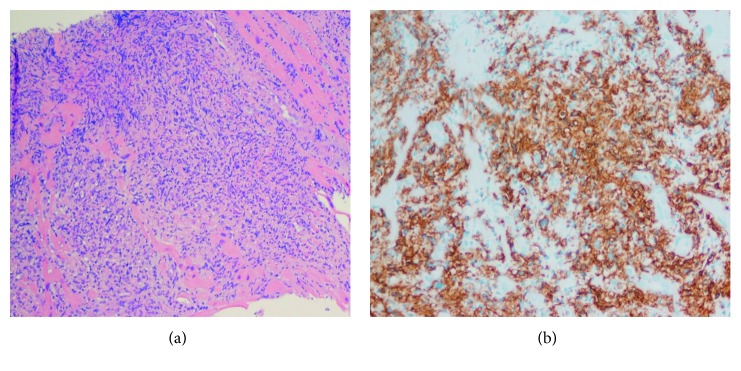
(a) H&E section of cardiac biopsy showing diffuse infiltration of large cells. (b) Immunohistochemistry staining positive for CD20.

**Figure 3:**
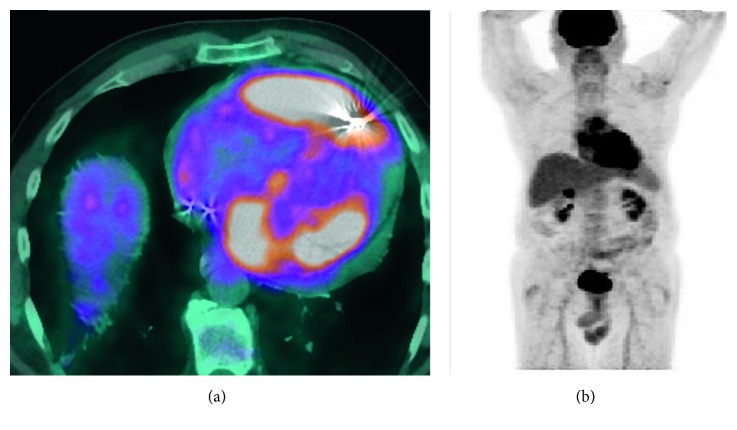
PET scan showing (a) increased FDG uptake in the cardiac tissue and (b) no extra cardiac FDG uptake.

A 69-year-old Caucasian male presented to the hospital with new onset dyspnea on exertion on walking 50 feet for past 3 weeks. Past medical history included hypertension, hyperlipidemia, atrial fibrillation of 2-month duration, and complete atrioventricular block status after permanent pacemaker placement a year preceding to current presentation. His home medications included lisinopril, metoprolol, apixiban, and atorvastatin. His laboratory work-up on presentation was unremarkable except mild elevation of uric acid at 8.5 mg/dl. HIV status was checked and was negative. Transthoracic echocardiography revealed pericardial effusion with evidence of pericardial tamponade and right ventricular wall hypertrophy. Pericardial window was performed, and pericardial fluid cytology was negative for any malignant cells. The patient was eventually discharged and referred to a heart failure specialist due to concerns for cardiac amyloidosis based on the right ventricular hypertrophy and conduction disease. A cardiac MRI was performed and showed a large mass, involving right ventricular (RV) lateral wall with a maximum thickness of 3 cm. Mass was hyperintense to myocardium on T2 and isointense on T1 (Figure 1). Left ventricular ejection fraction (EF) calculated using cardiac MRI was 41–43%. Cardiac biopsy of the RV mass was performed using an endovascular approach via the right internal jugular vein in the cardiac catheterization lab, assisted by intracardiac echocardiography. Additional work-up at that time included a coronary angiogram that showed absence of obstructive coronary disease. Immunohistochemistry (IHC) markers on the mass were positive for CD45, CD20, PAX-5, BCL2, BCL6, and MUM-1 and negative for CD5, CD10, and cyclin D1 (Figure 2). Ki-67 on the mass was 50–60%; EBER was negative along with FISH for MYC, BCL2, and BCL6. Findings from IHC were consistent with diffuse large B-cell lymphoma, nongerminal center subtype. Bone marrow biopsy performed as staging work-up was negative for any lymphoma involvement. The PET scan showed increased FDG (F-18 fluorodeoxyglucose) uptake in right atrium, right ventricle, and left ventricle with no abnormal uptake outside of the heart (Figure 3). The patient after getting a transthoracic echocardiography which confirmed EF at 45% was started on dose-adjusted R-EPOCH with 20% dose reduction in doxorubicin dose for the first cycle. In addition, he was noted to be chronically RV paced (99%) on pacemaker interrogation. It was decided that he may have had cardiomyopathy due to the RV pacing, and thus, he was upgraded to a biventricular pacemaker. After tolerating the first cycle, the patient was given full-dose doxorubicin starting the 2nd cycle. The interim PET scan after 2 cycles of R-EPOCH showed complete response (CR). The patient subsequently received 4 more cycles of R-EPOCH and continues to be in CR (confirmed by PET scan) 18 months after treatment. The patient also received dexrazoxane with 2nd, 3rd, and 4th cycles of chemotherapy to reduce cardiotoxicity of doxorubicin, given his existing cardiomyopathy. After chemotherapy, the patient's left ventricular EF is stable at 47% along with improvement in his atrial fibrillation. He remains on surveillance with 6 monthly echocardiography with a plan of getting cardiac MRI at 2 years after treatment completion.

## 3. Discussion

PCL is a rare disease and is less frequent than secondary cardiac involvement by lymphoma [6]. Most of the information comes from case reports and case series. Immune suppression by HIV is a major risk factor [7]. Petrich et al. [4] analyzed 197 cases of PCL described in the literature from 1949 to 2009. Immune status was only known for 64 cases, out of which 41% had HIV. The median age of PCL presentation is 63 years with male to female predominance by 2 : 1 [4]. Most common presenting signs/symptoms of PCL are nonspecific, including dyspnea, pericardial effusion, and arrhythmia [4, 8]. Echocardiography is important for diagnosis of PCL and has improved ability to detect cases antemortem, as before the first use of echocardiography in 1981 for PCL 64% of cases were at postmortem which has gone to 15% [4]. Cardiac MRI is an important diagnostic technique as it helps to differentiate PCL from other causes of primary cardiac masses such as sarcoma. Lymphoma appears hypo- or isointense on T1-weighted imaging or iso- or hyperintense on T2-weighted imaging [9]. PET scan is also important in diagnosis as well as following outcomes after treatment [10]. The right atrium is the most common site affected by PCL [4, 8]. The most common histological subtype for PCL is diffuse large-cell lymphoma, consisting of 72% of total PCL cases in SEER database analysis and 77% in analysis of 197 cases of PCL from 1949–2009 by Petrich et al. [4, 5]. Treatment mainly consists of anthracycline-based chemotherapy CHOP (cyclophosphamide, doxorubicin, vincristine, and prednisone) plus rituximab (anti-CD20 monoclonal antibody) [4]. Overall response rate (ORR) to chemotherapy is high, in analysis by Petrich et al. ORR to chemotherapy was 79% with 59% achieving CR, and in a single-center retrospective study by Carras et al., the ORR to chemotherapy was 85% with 62% of the patients achieving CR. Heart failure and sepsis remain two major causes of mortality [4]. As there is a risk of cardiac wall rupture with chemotherapy [11], for our patient, we used dose-adjusted R-EPOCH with 20% dose reduction in the first cycle. In R-EPOCH, doxorubicin is given in as an infusion over 72 hours and is less cardiotoxic compared to bolus doxorubicin given in R-CHOP [12]. To our knowledge, there are only two other published cases of PCL treated using R-EPOCH; in both cases, patients were able to achieve CR after only 2 cycles of chemotherapy (Table 1) [13, 14]. Our patient also received IV dexrazoxane with 2nd, 3rd, and 4th cycle in an attempt to reduce cardiotoxicity. Dexrazoxane has been shown to be cardioprotective without compromising the efficacy of chemotherapy and increasing the rate of secondary malignancies [15, 16]. Till date, there has been no published report of dexrazoxane use in PCL. Although more studies are needed to quantify benefit of using R-EPOCH plus dexrazoxane in PCL cases, these studies are difficult to complete due to low number of cases each year.

## 4. Conclusion

PCL is a rare malignancy with nonspecific presenting signs and symptoms. R-EPOCH should be used as front-line treatment for PCL due to less cardiotoxicity. Addition of dexrazoxane in the treatment strategy for PCL needs to be explored further.

## Figures and Tables

**Table 1:** Previous publications using R-EPOCH in PCL.

Publication	Patient age	Presenting signs/symptoms	Site of disease	Response to R-EPOCH	Follow-up
Rogers et al. [13]	72 years	Palpitations, dyspnea, dizziness	Right atrium, right ventricle	CR after 2 cycles	Chemotherapy discontinued after 3 cycles due to decline in performance status
Thiagraj et al. [14]	50 years	Abdominal pain, thromboembolism, pericardial tamponade	Left ventricle	CR after 2 cycles	Completed 6 cycles of chemotherapy, remains in CR at 1-year follow-up

## References

[1] Jeudy J., Kirsch J., Tavora F. (2012). From the radiologic pathology archives: cardiac lymphoma: radiologic-pathologic correlation. *Radiographics*.

[2] Whorton C. M. (1949). Primary malignant tumors of the heart report of a case. *Cancer*.

[3] Maleszweski J. J., Jaffe E. S., Travis W. D., Marx A., Barambilla E., Nicholson A., Burke A. (2015). Cardiac lymphomas. *WHO Classification of Tumors of the Lung, Pleura, Thymus and Heart*.

[4] Petrich A., Cho S. I., Billett H. (2011). Primary cardiac lymphoma. *Cancer*.

[5] Oliveira G. H., Al-Kindi S. G., Hoimes C., Park S. J. (2015). Characteristics and survival of malignant cardiac tumors: a 40-year analysis of over 500 patients. *Circulation*.

[6] Grebenc M. L., Rosado de Christenson M. L., Burke A. P., Green C. E., Galvin J. R. (2000). Primary cardiac and pericardial neoplasms: radiologic-pathologic correlation. *Radiographics*.

[7] Guarner J., Brynes R. K., Chan W. C., Birdsong G., Hertzler G. (1987). Primary non-Hodgkin’s lymphoma of the heart in two patients with the acquired immunodeficiency syndrome. *Archives of Pathology & Laboratory Medicine*.

[8] Carras S., Berger F., Chalabreysse L. (2017). Primary cardiac lymphoma: diagnosis, treatment and outcome in a modern series. *Hematological Oncology*.

[9] Luna A., Ribes R., Caro P., Vida J., Erasmus J. J. (2005). Evaluation of cardiac tumors with magnetic resonance imaging. *European Radiology*.

[10] Castelli J. B., Alexandre L., Futuro G., Scanavacca M., Soares Júnior J. (2011). Primary cardiac lymphoma detected by 18F-FDG PET scan: a case report. *Journal of Nuclear Cardiology*.

[11] O’mahony D., Piekarz R. L., Bandettini W. P., Arai A. E., Wilson W. H., Bates S. E. (2008). Cardiac involvement with lymphoma: a review of the literature. *Clinical Lymphoma and Myeloma*.

[12] Legha S. S. (1982). Reduction of doxorubicin cardiotoxicity by prolonged continuous intravenous infusion. *Annals of Internal Medicine*.

[13] Rogers S. C., O’Connor O. A., Granati G., Yaddanapudi K. (2016). The first case of primary cardiac lymphoma, diffuse large B-cell type, successfully treated with EPOCH-R. *Blood*.

[14] Thiagaraj A., Kalamkar P., Rahman R., Farah V., Poornima I. (2018). An unprecedented case report of primary cardiac lymphoma exclusive to left ventricle: a diagnostic and therapeutic challenge. *European Heart Journal-Case Reports*.

[15] Asselin B. L., Devidas M., Chen L. (2016). Cardioprotection and safety of dexrazoxane in patients treated for newly diagnosed T-cell acute lymphoblastic leukemia or advanced-stage lymphoblastic non-hodgkin lymphoma: a report of the children’s oncology group randomized trial pediatric oncology group 9404. *Journal of Clinical Oncology*.

[16] Swain S. M., Whaley F. S., Gerber M. C. (1997). Cardioprotection with dexrazoxane for doxorubicin-containing therapy in advanced breast cancer. *Journal of Clinical Oncology*.

